# Utilizing multi-trait selection indices for genetic characterization and SSR marker validation linked to powdery mildew resistance in sunflower

**DOI:** 10.1038/s41598-026-52132-3

**Published:** 2026-05-27

**Authors:** Venkatesh Gopi, Lavudya Sampath, Kalaimagal Thiyagarajan, Sasikala Ramasamy, Sankarasubramanian Harish, Boya Nagendra Naidu, Bonipas Antony John, Dhamotharan Palanisamy

**Affiliations:** 1 Department of Genetics and Plant Breeding, CPBG, TNAU, Coimbatore, Tamil Nadu 641 003 India; 2 Department of Oilseeds, CPBG, TNAU, Coimbatore, Tamil Nadu 641003 India; 3 Department of Plant Pathology, TNAU, Coimbatore, Tamil Nadu 641003, India

**Keywords:** FAI-BLUP, GYT, MGIDI, PCoA, Population structure, Powdery mildew, Biotechnology, Genetics, Plant sciences

## Abstract

In india sunflower, an important crucial oilseed crop, has endured considerable yield declines over the past two decades. One of the factor in the vulnerability of the reduced cultivation to powdery mildew disease. This persistent challenge underscores the pressing necessity for resistant germplasm and robust molecular markers to advance breeding initiatives. In this study, the phenotypic diversity of 20 sunflower inbreds was assessed for traits during two seasons (winter 2022, rainy season 2023). Using three multi trait selection indices Genotype-by-Yield-Trait (GYT) biplot, the Multi-Trait Genotype-Ideotype Distance Index (MGIDI) and the Factor Analysis and Interaction Best Linear Unbiased Prediction (FAI-BLUP) were used to evaluate the inbred lines. The inbreds were screened for powdery mildew resistance under both natural (winter 2022, rainy season 2023) and artificial conditions (summer 2023). Significant variation was observed for all traits, with four inbreds (COSF 6B, COSF 10B, COSF 12B and COSF 15B) exhibiting high oil content, seed yield and oil yield per plant. The inbreds COSF 6B, CSFI-17008 and CSFI-17024 consistently achieved the highest rankings across all indices, demonstrating strong potential for exploitation breeding programmes. SSR marker-based cluster analysis using DARwin software grouped the inbreds into five distinct clusters and STRUCTURE analysis revealed five sub-populations. Out of 141 SSR markers tested, 92 primers were polymorphic, with ORS898, ORS188, ORS1008 and ORS725 identified as the most effective. Marker validation using ORS 684 and ORS1110, both linked to powdery mildew resistance, showed that ORS 684 explained 27.17% of phenotypic variation and reliably distinguished resistant and susceptible inbreds. Notably, CSFI 17018 and CSFI 17020 exhibited resistance under both screening conditions and their resistance was further confirmed by marker validation. Based on diversity analyses and artificial screening, we recommend utilizing the identified diverse inbreds-COSF 6B, COSF 10B, COSF 12B, COSF 15B, CSFI 13023, CSFI 17008, CSFI 17018 and CSFI 17020-as parental lines in future breeding programs. For developing powdery mildew-resistant hybrids, inbreds, or varieties through marker-assisted backcrossing, ORS 684 is proposed as an effective foreground marker to facilitate the transfer of resistance loci.

**Supplementary Information:**

The online version contains supplementary material available at 10.1038/s41598-026-52132-3.

## Introduction

Sunflower (*Helianthus annuus* L.), a cross-pollinated crop of the Asteraceae family, is widely cultivated for its high oil content (22–50%), drought tolerance, short growing duration and adaptability to a broad range of agro-climatic conditions^1–3^. As an important source of vegetable oil and a valuable crop for both human consumption and industrial applications, sunflower contributes significantly to the global edible oil supply. The Food and Agriculture Organization (FAO) estimates that global demand for sunflower oil will increase to 60 million tons by 2050^4^, imposing an urgent need for sunflower breeding programs to focus on developing inbreds and hybrids that combine high seed yield, superior oil quality and resilience to both biotic and abiotic stresses.

The foundation of hybrid development lies in the selection of genetically diverse inbreds, which serve as parents to maximize heterotic potential in segregating generations. Phenotypic and molecular characterization of inbreds is critical for this purpose, as it provides insights into genetic variability and potential performance^5^. However, selection of inbreds is challenged by complex trait correlations, significant genotype-by-environment interactions and the inherent difficulty of balancing multiple, often antagonistic traits such as yield, oil quality and disease resistance. Traditional selection indices, such as the Smith-Hazel index, are constrained by subjective weighting of traits, multicollinearity and the requirement for extensive, balanced datasets, limiting their applicability in practical breeding programs^6,7^.

To overcome these limitations, advanced multivariate approaches have been developed to facilitate the objective evaluation of genotypes across multiple traits. The Genotype-by-Yield*Trait (GYT) biplot method^8^ integrates yield and other important agronomic traits into a unified analytical framework, enabling breeders to visually identify superior inbreds based on their combined performance. This method is particularly useful when selecting for disease resistance traits such as Percent Disease Index (PDI) for powdery mildew, as it allows direct comparison of genotypes under both disease and yield criteria. Similarly, the Factor Analysis and Ideotype-BLUP (FAI-BLUP) method integrates factor analysis with Best Linear Unbiased Prediction (BLUP) models, enabling the simultaneous evaluation of multiple correlated traits and comparison against an ideal target phenotype defined by the breeder^9^. Similarly, the Multi-Trait Genotype-Ideotype Distance Index (MGIDI) ranks genotypes based on their Euclidean distance from a pre-specified ideotype, effectively managing multicollinearity and environmental effects to provide a reliable and interpretable selection criterion^10^. These advanced indices have been successfully applied across several important crops, demonstrating their robustness in complex breeding scenarios^11–13^.

Among the major biotic stresses affecting sunflower production, powdery mildew-caused by *Golovinomyces latisporus* L. is one of the most destructive, causing yield and oil quality losses of 30–74%^14,15^. The disease is particularly problematic in tropical and subtropical regions with high humidity, where its rapid spread can devastate large crop areas. While chemical fungicides are widely applied for disease control, they are associated with high costs, potential health hazards, environmental pollution and the risk of developing fungicide-resistant pathogen strains. Therefore, the development of inbreds with innate resistance to powdery mildew is a sustainable, economically viable and environmentally sound solution. Unfortunately, only limited research has identified QTLs or molecular markers linked to powdery mildew resistance in sunflower^16–18^. Moreover, validation of these markers across diverse genetic backgrounds is critical before implementing marker-assisted selection (MAS) strategies in breeding programs. Among the few reported markers, SSR markers ORS 684 and ORS 1110 have been associated with powdery mildew resistance loci^18^, but further validation is required for wider application.

Additionally, assessment of genetic diversity solely based on agro-morphological traits is often unreliable due to their strong environmental influence, leading to inconsistent or misleading results^2,19,20^. In contrast, DNA-based markers such as SSRs offer stable polymorphism, high reproducibility and independence from environmental factors, making them highly suitable for diversity analysis, population structure assessment and linkage mapping^2,21–23^.

Therefore, in light of these challenges and the need for efficient breeding strategies, the present study was undertaken with the following objectives: (i) to evaluate phenotypic and molecular diversity among 20 sunflower inbreds, (ii) to apply advanced multi-trait selection indices (GYT biplot, FAI-BLUP and MGIDI) for integrated selection of inbreds based on yield, oil quality and powdery mildew resistance under both artificial and natural infection conditions and (iii) to validate previously reported SSR markers ORS 684 and ORS 1110 associated with powdery mildew resistance, thereby contributing valuable tools for future marker-assisted breeding efforts.

## Materials and methods

### Location and experimental materials

The study was carried out at the Department of Oilseeds, Centre for Plant Breeding and Genetics (CPBG), Tamil Nadu Agricultural University, Coimbatore, situated at an elevation of 426.72 m (11° N latitude, 77° E longitude). Weather conditions during the experiment season given in Supplementary Table 1. Twenty inbreds with different genetic backgrounds, including B (maintainer) and R (restorer) lines, were selected and sown during winter (*rabi)* 2022 and rainy (*kharif* ) 2023 seasons in a randomized block design (RBD) with three replications. These inbreds collected from Tamil Nadu Agriculture University, Coimbatore (14), University of Agricultural Sciences, Bangalore (3), University of Agricultural Sciences, Raichur (2) and Indian Institue of Oilseeds Research, Hyderabad (1), are listed in Supplementary Table 2. Each inbred was planted in 5 m rows with 60 × 30 cm spacing, following standard agronomic practices.

### Morphological characterization and estimation of oil content using NIR spectrometer

Data was collected from five randomly selected plants per accession and characterized for ten traits, which includes: days to 50% flowering (DF), days to maturity (DM), plant height (PH) (cm), head diameter (HD) (cm), 100- seed weight (HSW) (g), volume weight per 100 ml (VW) (g), oil content (OC) (%), seed yield per plant (SYP) (g), oil yield per plant (OYP) (g) and percent disease index (PDI) of powdery mildew. Oil content was estimated using a NIR spectrometer (ZEUTEC, Germany; Model: SPA 1.0), referencing an established standard graph. Calibration involved grinding 5-6 g of sunflower seeds from each sample into a fine powder using a mixer grinder for 1 to 2 min. The oil content was expressed as a percentage^24,25^.

$${\mathrm{Oil}}\;{\mathrm{yield}}\;{\mathrm{per}}\;{\text{plant }}\left( {\mathrm{g}} \right) = \frac{{{\mathrm{seed}}\;{\mathrm{yield}}\;{\mathrm{per}}\;{\text{plant }}\left( {\mathrm{g}} \right) \times {\mathrm{oil}}\;{\text{content }}\left( {{\% }} \right)}}{100}.$$

### DNA extraction and SSR markers

A total of 141 sunflower SSR markers, distributed across all the 17 linkage groups, were selected based on Tang et al.^26^. DNA was extracted from 14-day old seedlings of 20 inbreds in *summer* season 2023, using the CTAB method^27^. The DNA was checked on 0.8% agarose gel electrophoresis, quantified with a nanoquant ND-1000 (Thermo, USA) and normalized to 10 ng/μl. PCR reactions consisted of 2 µl DNA, 1 µl of primers (0.5 µl of forward and reverse primer each), 3 µl master mix and 4 µl sterile water for a total volume of 10 µl. Annealing temperatures were adopted from Hube et al.^28^. PCR products were visualized on a 3% agarose gel and documented with the GELSTAIN (M/s Medicare, India).

### Molecular characterization of the pathogen

**Fig. 1:**
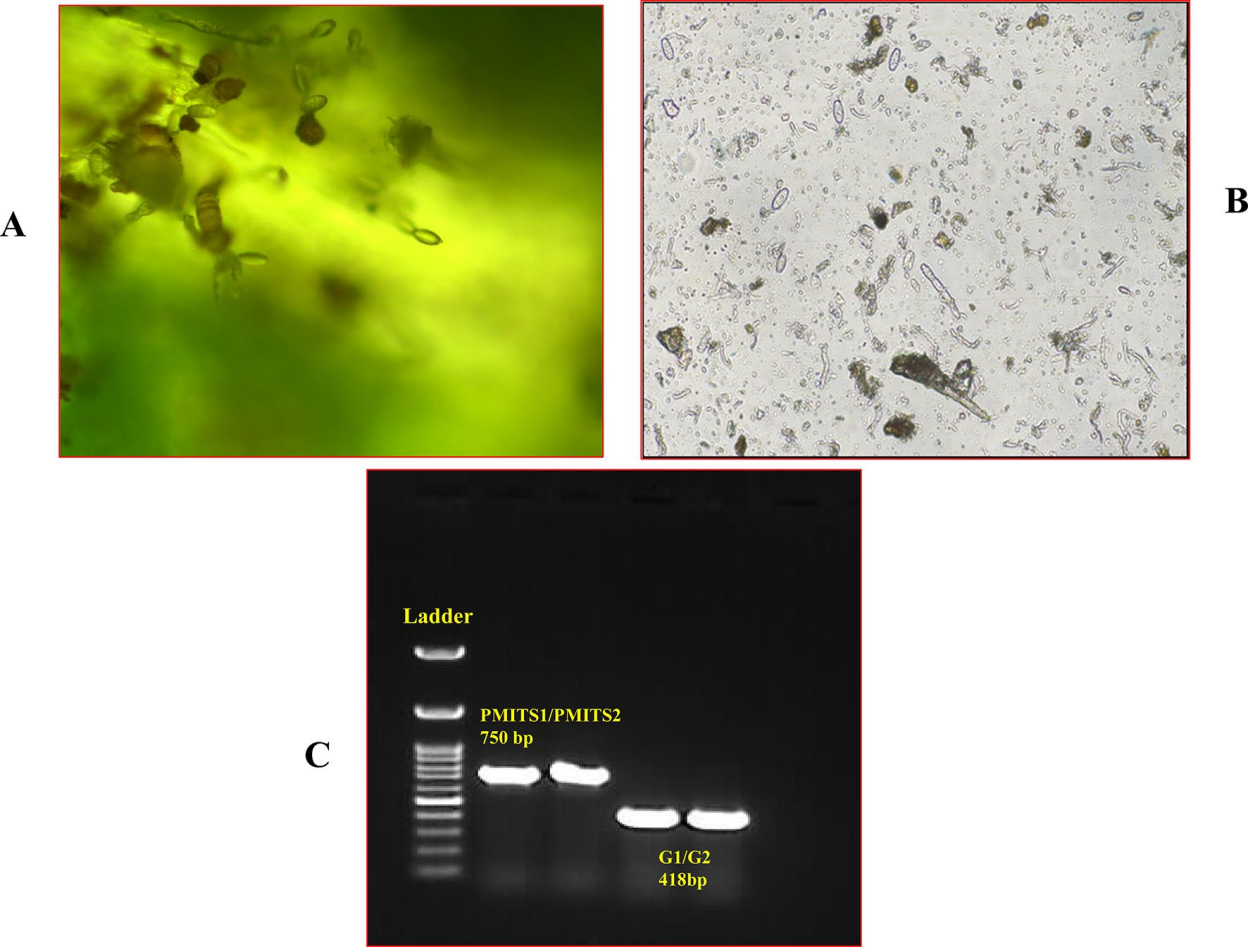
Confirmation of powdery mildew pathpgen in sunflower. (**A**) Chains of conidia observed on leaves using phase contrast microscopy; (**B**) Conidia visualized in sucrose suspension with phase contrast microscopy; (**C**) PCR analysis confirming powdery mildew with specific primers-PMITS1/PMITS2 producing a 750 bp fungal band and G1/G2 yielding a 418 bp band specific for *Golovinomyces latisporus.*

Prior to initiating the screening program, it is essential to confirm the causal pathogen using microscopic and/or molecular methods. In this study, the presence of the specific powdery mildew pathogen was confirmed through PCR using Internal Transcribed Spacer (ITS) and species-specific primers^18,29^. DNA was extracted from the conidia on the infected leaves using the CTAB method. PCR was performed using the PMITS primer to detect powdery mildew fungi (Erysiphales)^30^ and the G1/G2^18,29^ primers for identifying *G. latisporus*. Additionally, conidia were scraped from infected leaves and examined under a phase contrast microscope (Leica DM2000, Germany) (Fig. 1A,B). The presence of spores in the inoculam was confirmed through the observation of barrel-shaped conidia. PCR amplification was performed following the procedures of Chen et al.^31^ and Rajeshwaran et al.^18^, with the amplified products analyzed using 2% agarose gel electrophoresis.

### Screening for powdery mildew resistance under natural and artificial conditions

Screening of the inbreds for reaction to powdery mildew was conducted under natural field condition during winter 2022 and rainy season 2023 and artificial inoculated condition during *summer* 2023. The percent disease index (PDI) was calculated at 45, 60 and 75 days after sowing (DAS) using the standard 0-9 scale described by Mayee and Datar^32^, following the method outlined by McKinney^33^. For artificial screening, the inbreds were raised in pots, initially planted with three seedlings per pot and later thinned to one seedling per pot. Three pots were maintained for each inbred. The resistant lines IR6 and PM 81 and the susceptible line COSF 15B were used for comparison. Artificial inoculation was carried out by spraying a conidial suspension (1 × 10⁶ CFU/mL) of *G. latisporus* prepared by immersing powdery mildew-infected leaves in 1% sucrose solution, onto 35-day-old seedlings of each inbred. The application was done twice, at 3-4 day intervals, in the morning and evening.

$${\mathrm{PDI}} = \frac{{{\mathrm{Summation}}\;{\mathrm{of}}\;{\mathrm{all}}\;{\mathrm{numerical}}\;{\mathrm{ratings}}}}{{{\mathrm{Total}}\;{\mathrm{number}}\;{\mathrm{of}}\;{\mathrm{leaves}}\;{\text{observed }} \times {\text{ maximum }}\;{\text{rating }}}} \times 100.$$

### Validation of powdery mildew specific markers

The presence of powdery mildew resistant QTL was validated in the 20 inbreds using specific markers (ORS 684 and ORS 1110) as reported by Rajeshwaran et al.^18^. These markers validated the presence of QTL to assess their effectiveness in different genetic backgrounds. Phenotypic data were based on PDI and the genotypic data were derived from co-dominant marker coding. Single marker analysis used phenotypic data as the dependent variable and marker data as the independent variable. Marker validation was conducted using standard regression analysis in Microsoft Excel.

### Statistical analysis

Each SSR band was scored as a dominant marker (1 for presence, 0 for absence) and treated as an independent locus. Binary data were used to compute the Polymorphism Information Content (PIC) and Resolving Power (Rp) according to Sharma et al.^34^ and multiplex ratio and marker index were calculated following Powell et al.^35^. Allelic data were used to assess genetic distances and neighbor-joining tree was constructed with DARwin 6.0 software package^36^. Analysis of molecular variance (AMOVA) and Principal Coordinate Analysis (PCoA) were performed using GenAlEx V6.5^37^. Population structure (PS) was evaluated using STRUCTURE 2.3.4^38^ with five replications per K value, each including 100,000 burn-in steps and 100,000 Monte Carlo Markov Chain replicates. Inbreds membership was evaluated across K = 1 to 8 using the admixture model with associated allele frequencies. The optimal K was determined by analyzing ΔK values from the LnPD graph and the final population structure was established with “StructureSelector” software^39–41^.

Morphological and yield-related data were subjected to analysis of variance (ANOVA) for each season, followed by pooled ANOVA across seasons to assess significant variation among inbreds. All subsequent analyses were performed using pooled data. Best linear unbiased predictors (BLUPs) were estimated using the restricted maximum likelihood (REML) method with the R package *metan*^42^ and these BLUP values were subsequently used for clustering inbreds via the Ward method implemented in *factoextra*. Multi-trait selection was performed using three complementary approaches: the Genotype-by-Yield*Trait (GYT) biplot, the Multi-Trait Genotype-Ideotype Distance Index (MGIDI) and Factor Analysis and Ideotype-based BLUP (FAI-BLUP). In the GYT biplot, yield-trait combinations were calculated by multiplying oil yield per plant (OYP) with traits where higher values are desirable and dividing OYP by traits where lower values are preferable. The resulting combined values were standardized (mean = 0, standard deviation = 1) and summed to obtain a GYT index for each inbred, with the biplot constructed using the first two principal components derived from singular value decomposition (scaling = 1, centering = 2, singular value partitioning = 2)^8^. In MGIDI, all traits were rescaled to a uniform 0-100 range according to the desired selection direction and factor analysis was performed to reduce dimensionality and account for correlations among traits, followed by calculation of Euclidean distances from a predefined ideotype to rank inbreds^43^. Similarly, FAI-BLUP incorporated BLUPs and factor analysis to group correlated traits, with genotypes ranked based on their distance from an ideotype defined by desired trait values^9^. All analyses were conducted in R (version 4.3.0), with visualization of biplots, genotype rankings and factor contributions performed using the *metan* package, allowing robust and objective evaluation of sunflower inbreds across yield, oil quality and disease resistance traits.

## Results

### Pooled analysis of variance, mean performance and cluster analysis of inbreds based on morphological traits

Analysis of variance for both the winter 2022 and rainy 2023 seasons revealed significant differences among the inbreds. The pooled ANOVA confirmed significant effects of season and the inbreds × season interactions for all the traits examined (Supplementary Table 3). The pooled mean performance formed the basis for selecting superior inbreds. Notably, COSF 6B, COSF 12B and IR6 exhibited the highest oil content, while COSF 6B, COSF 12B, COSF 15B and CMS 302B had high seed yield per plant. Furthermore, COSF 12B and COSF 6B were top performers for oil yield per plant when averaging across two seasons among the 20 inbreds.

**Fig. 2:**
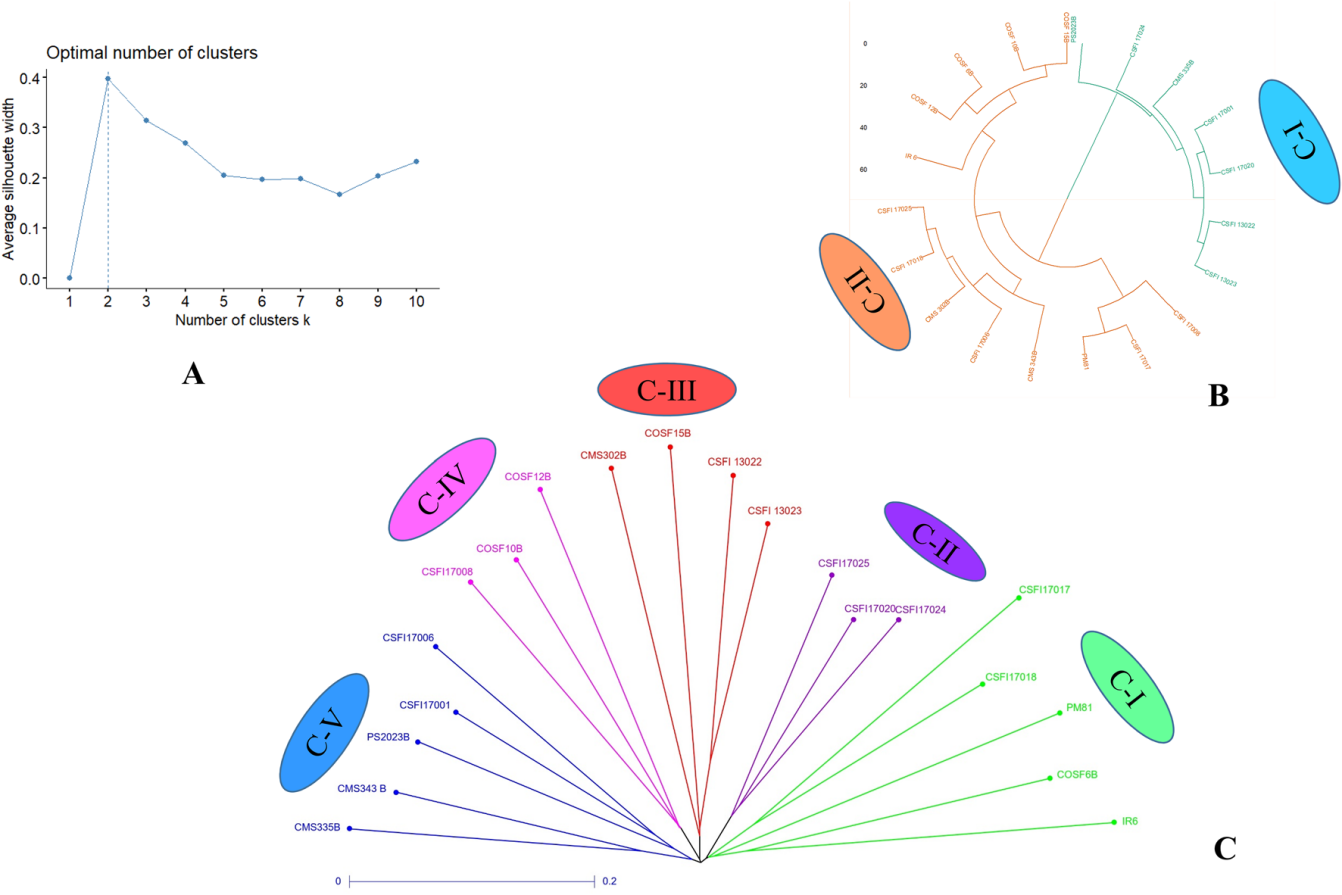
Clustering of 20 sunflower inbred lines. (**B**) Grouping based on morphological traits into two clusters; (**C**) Neighbor-joining tree constructed using DARwin software with data from 92 SSR markers; (**A**) Determination of the optimal number of clusters by the Elbow Method using morphological data.

Cluster analysis was performed using Ward’s method with Euclidean distance as the similarity metric. The Elbow method indicated two as the optimal number of clusters. Accordingly, Cluster I comprised seven genotypes, while Cluster II included 13 genotypes (Fig. 2A,B).

### Multi-trait selection using Genotype × Yield × Trait (GYT) biplots

**Fig. 3:**
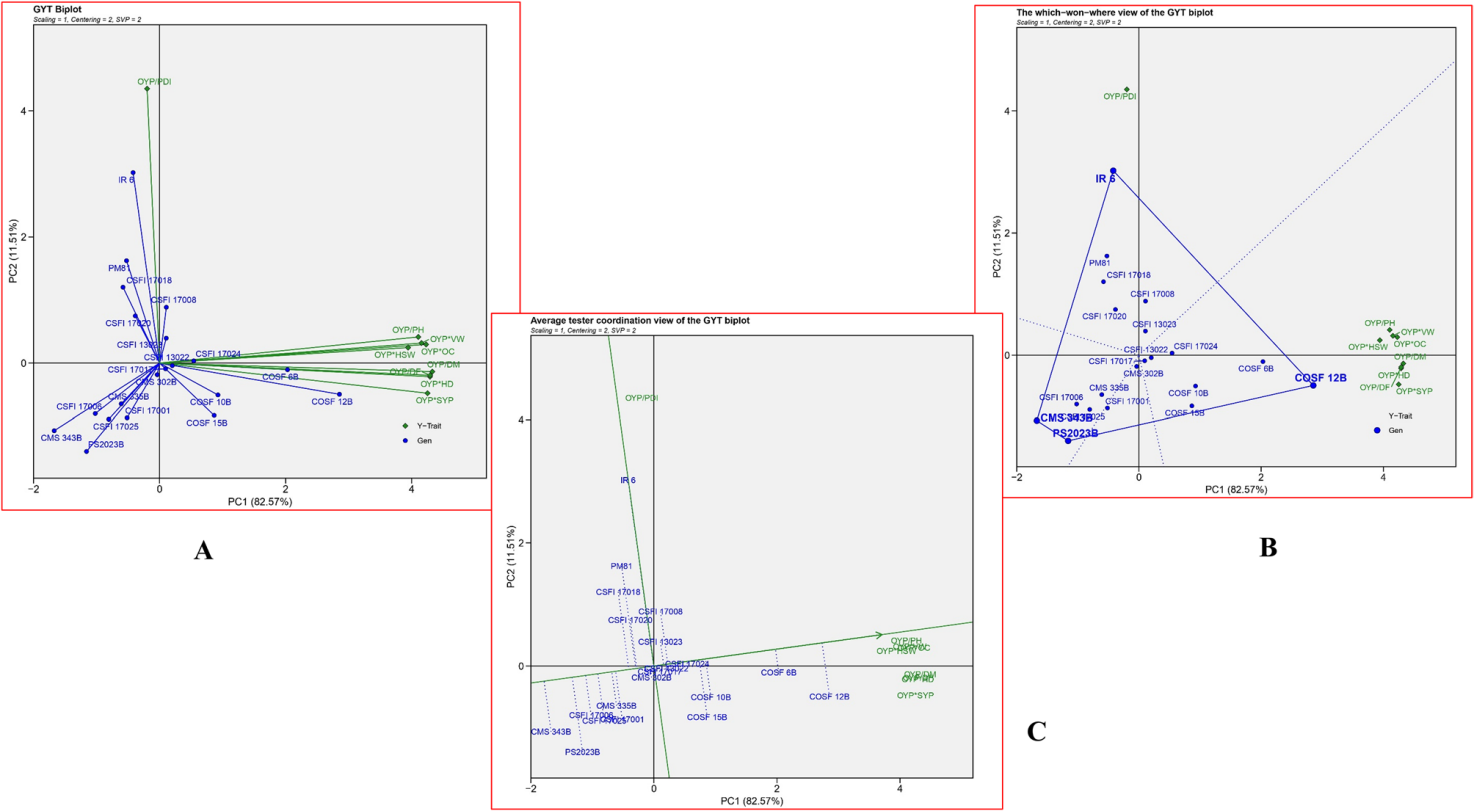
Visualization of genotype performance using GYT biplot analysis. (**A**) GYT biplot illustrating relationships among yield-trait combinations, generated by singular value decomposition (SVD) of the standardized GYT matrix with scaling = 1, centering = 2 and trait-focused singular value partition (SVP) = 2; (**B**) "Which-won-where" view highlighting genotypes with superior performance profiles; (**C**) Average tester coordination (ATC) view comparing and ranking lines by trait profiles, based on the same SVD parameters.

The GYT biplot was constructed using the first two principal components (PC1 and PC2), which together explained 94.08% of the total variation. It revealed strong positive correlations among most yield-trait indices, except for OYP/PDI, which showed moderate correlations with OYP/PH, OYP*VW, OYP*OC and OYP*HSW (Fig. 3A). In the "which-won-where" polygon view, yield–trait combinations were segmented into sectors by perpendicular lines, facilitating the identification of genotypes performing best for specific trait combinations. The inbred COSF 12B consistently outperformed other inbreds across most yield–trait indices, except OYP/PDI. IR6 excelled specifically for OYP/PDI, highlighting its ability to combine powdery mildew resistance with yield potential. PS 2023B and CMS 343B did not demonstrate superiority in any evaluated trait combination (Fig. 3B). The average tester axis (ATA), shown as a single-arrow line passing through the biplot origin, indicated the direction of increasing mean performance across all yield–trait combinations. Genotypes positioned to the right of the ATA were considered superior, while those to the left were below average (Fig. 3C). A perpendicular double-arrow line further divided the biplot into regions, clearly separating above-average genotypes from below-average ones. Based on the ATA and standardized GYT index scores, (Table 1) which capture each genotype’s strengths and weaknesses across traits, the top five ranked inbreds were COSF 12B, COSF 6B, COSF 10B, COSF 15B and CSFI 17024. The lowest-ranking inbreds included CSFI 17018, CSFI 17025, PS2023B, CSFI 17006 and CMS 343B. In the genotype ranking view, the best-performing inbreds clustered within the innermost concentric circle, indicating their robust multi-trait performance.

**Table 1:** The data of standardized genotype by yield*trait (GYT), superiority index and MGIDI for sunflower inbreds.

S.No	Gen	OYP*HD	OYP*SYP	OYP*HSW	OYP*VW	OYP*OC	OYP/PH	OYP/DF	OYP/DM	OYP/PDI	coincidence	Rank	MGIDI
1	COSF 12B	2.917	5.362	− 1.085	9.196	9.698	− 2.365	− 2.361	− 2.363	− 2.356	16.64	1	4.06
2	COSF 6B	2.074	3.436	− 1.205	6.650	8.265	− 2.365	− 2.362	− 2.364	− 2.354	9.78	2	3.35
3	COSF 10B	0.978	1.545	− 1.450	5.669	5.762	− 2.365	− 2.363	− 2.364	− 2.357	3.05	3	3.69
4	COSF 15B	0.612	2.010	− 1.651	4.322	5.972	− 2.365	− 2.363	− 2.364	− 2.359	1.82	4	5.23
5	CSFI 17024	0.534	0.908	− 1.389	5.271	4.579	− 2.366	− 2.363	− 2.365	− 2.352	0.46	5	3.34
6	CSFI 13022	− 0.237	0.988	− 1.520	4.538	4.109	− 2.366	− 2.364	− 2.365	− 2.353	− 1.57	6	4.48
7	CSFI 13023	0.103	0.602	− 1.471	4.715	3.192	− 2.366	− 2.364	− 2.365	− 2.350	− 2.30	7	3.67
8	CSFI 17017	0.112	0.499	− 1.641	4.097	3.106	− 2.365	− 2.364	− 2.365	− 2.354	− 3.28	8	3.20
9	IR 6	− 0.436	− 0.471	− 1.804	3.662	3.893	− 2.366	− 2.365	− 2.365	− 2.330	− 4.58	9	5.10
10	CMS 302B	− 0.149	1.325	− 1.544	3.103	2.027	− 2.365	− 2.364	− 2.365	− 2.353	− 4.69	10	4.42
11	CSFI 17008	− 0.197	0.179	− 1.475	2.306	3.142	− 2.365	− 2.364	− 2.365	− 2.346	− 5.49	11	3.40
12	CSFI 17020	− 0.166	− 0.168	− 1.537	2.537	2.293	− 2.366	− 2.364	− 2.365	− 2.347	− 6.48	12	4.02
13	PM81	− 0.588	− 0.447	− 1.892	2.676	2.821	− 2.366	− 2.364	− 2.365	− 2.340	− 6.87	13	4.28
14	CSFI 17001	− 0.582	− 0.168	− 1.674	2.558	2.390	− 2.366	− 2.365	− 2.365	− 2.359	− 6.93	14	5.01
15	CMS 335B	− 0.607	− 0.060	− 1.768	3.040	1.389	− 2.366	− 2.365	− 2.365	− 2.357	− 7.46	15	4.15
16	CSFI 17018	− 0.586	− 0.190	− 1.684	2.250	1.601	− 2.366	− 2.364	− 2.365	− 2.343	− 8.05	16	3.92
17	CSFI 17025	− 0.789	− 0.610	− 1.960	2.189	1.976	− 2.366	− 2.365	− 2.365	− 2.359	− 8.65	17	5.14
18	PS2023B	− 0.776	− 1.040	− 1.918	0.904	1.315	− 2.366	− 2.365	− 2.366	− 2.362	− 10.97	18	6.54
19	CSFI 17006	− 0.850	− 0.753	− 1.913	1.527	0.390	− 2.366	− 2.365	− 2.365	− 2.358	− 11.05	19	3.94
20	CMS 343B	− 1.367	− 1.417	− 1.987	− 0.467	− 0.450	− 2.366	− 2.366	− 2.366	− 2.359	− 15.15	20	6.09

*DF* Days to 50% flowering, *DM* Days to Maturity, *PH* Plant height, *HD* Head diameter, *HSW* Hundred Seed weight, *VW* Volume weight per 100 ml, *OC* Oil content, *SYP* Yield per plant, *OYP* Oil yield per plant, *PDI* Percent of disease index.

### Multi-trait selection using MGIDI and FAI-BLUP

In this study, inbred selection was further refined using two advanced multi-trait selection indices: the Multi-Trait Genotype-Ideotype Distance Index (MGIDI) and the Factor Analysis and Interaction Best Linear Unbiased Prediction (FAI-BLUP). Principal component analysis (PCA) performed on REML/BLUP data identified four principal components (PC1 to PC4) that together explained 84.04% of the total trait variation. Specifically, PC1 accounted for 40.34% of the variance, followed by PC2 (17.98%), PC3 (14.17%) and PC4 (11.55%). Varimax rotation was applied to optimize factor loadings, which enhanced interpretability by clearly grouping correlated traits and improving the precision of inbred selection toward an ideotype.

The communality values in both MGIDI and FAI-BLUP ranged from 0.74 (HD) to 0.94 (OYP) (Table 2), with an overall average communality of 0.84 across all traits. Traits were grouped into four factors: FA1 (HD, SYP, OC, OYP, PDI), FA2 (PH, DF, DM), FA3 (OC, PDI) and FA4 (PDI, DF, DM). Selected inbreds consistently exhibited higher average values than population means for HD, SYP, OYP, HSW and OC, indicating genetic gains in key agronomic and quality traits. Conversely, percent disease index (PDI), DF and DM showed negative selection differentials (SD%), reflecting favorable reductions in disease incidence and developmental duration, which are important for enhancing powdery mildew resistance and earliness.

**Table 2:** Selection differential and estimates for effective traits in the MGIDI index and FAI-BLUP.

S.No	FAI-BLUP								MGIDI							
	VAR	Factor	Xo	Xs	SD	SDperc	sense	goal	VAR	Factor	Xo	Xs	SD	SDperc	sense	goal
1	HD	1	14.11	15.35	1.25	8.85	I	100	HD	FA1	14.11	15.43	1.33	9.41	I	100
2	SYP	1	17.01	18.52	1.52	8.93	I	100	SYP	FA1	17.01	18.44	1.44	8.46	I	100
3	OYP	1	5.84	6.85	1.01	17.26	I	100	OYP	FA1	5.84	6.82	0.98	16.82	I	100
4	PDI	1	14.09	12.52	− 1.57	− 11.16	D	100	PDI	FA1	14.09	13.19	− 0.90	− 6.40	D	100
5	PH	2	135.75	137.38	1.63	1.20	D	0	PH	FA2	135.75	127.53	− 8.22	− 6.05	D	100
6	DF	2	59.45	54.13	− 5.33	− 8.96	D	100	DF	FA2	59.45	53.71	− 5.74	− 9.66	D	100
7	DM	2	90.72	86.50	− 4.22	− 4.65	D	100	DM	FA2	90.72	86.04	− 4.68	− 5.15	D	100
8	HSW	3	4.52	5.20	0.68	15.12	I	100	HSW	FA3	4.52	4.97	0.45	9.90	I	100
9	VW	4	35.55	37.06	1.50	4.22	I	100	VW	FA4	35.55	36.30	0.74	2.09	I	100
10	OC	4	33.94	36.73	2.78	8.20	I	100	OC	FA4	33.94	36.74	2.80	8.25	I	100

*Xo* overall mean of inbreds, *Xs* mean of the selected inbreds, *SD* selection differential, *DF* Days to 50% flowering, *DM* Days to Maturity, *PH* Plant height, *HD* Head diameter, *HSW* Hundred Seed weight, *VW* Volume weight per 100 ml, *OC* Oil content, *SYP* Yield per plant, *OYP* Oil yield per plant, *PDI* Percent of disease index.

### Selection intensity and inbreds ranking visualization

**Fig. 4:**
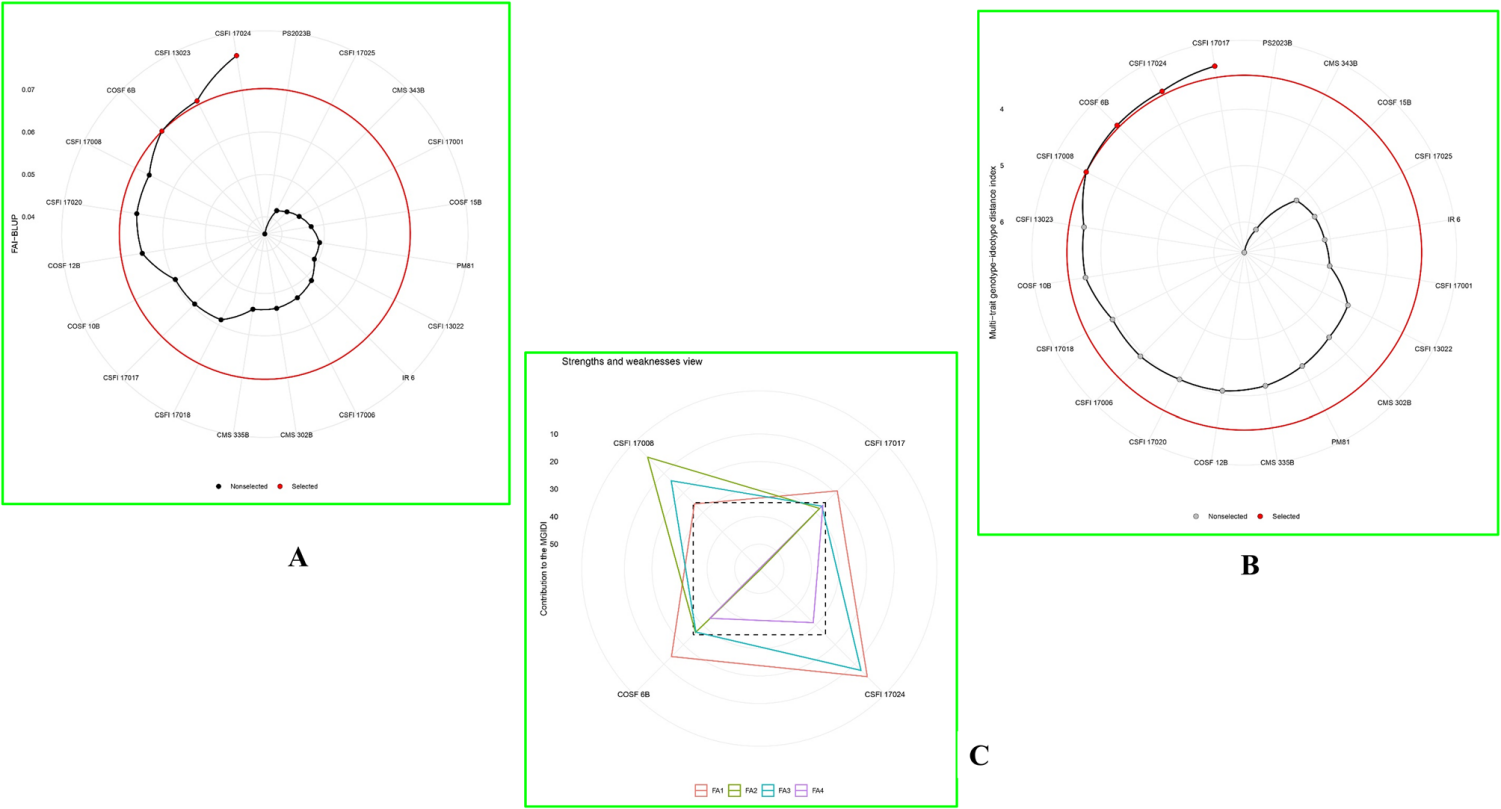
Inbred selection at 20% intensity. (**A**) Inbred ranking using the MGIDI index across traits, with selected inbreds highlighted in red and cut-off point shown; (**B**) Inbred ranking using the FAI-BLUP index across traits, with selected inbreds highlighted in red and cut-off point shown; (**C**) Strengths and weaknesses of selected inbreds depicted proportionally using the MGIDI index.

The MGIDI and FAI-BLUP indices were applied to comprehensively evaluate all measured traits and identify superior sunflower inbreds. Using a 20% selection intensity, the MGIDI-based ranking identified CSFI-17017 (3.20), CSFI-17024 (3.34), COSF 6B (3.35),and CSFI-17008 (3.40) as the top-performing inbreds, with MGIDI values ranging from 3.20 (CSFI-17017, best performer) to 6.54 (PS2023B, poorest performer) and an overall mean of 4.35 (Fig. 4A). The lowest-ranking inbreds included PS2023B (6.54), CMS 343B (6.09), COSF 15B (5.23) and CSFI 17025 (5.14). Similarly, the FAI-BLUP method identified CSFI-17024, CSFI-13023, COSF 6B and CSFI-17008 (Fig. 4B) as superior genotypes.

### Strength and weakness of inbreds based on MGIDI

Factor-specific contributions of the MGIDI index provided deeper insights into the strengths and weaknesses of individual inbreds by quantifying how each factor contributed to the overall genotype-ideotype distance. CSFI-17024 exhibited strong associations with FA1 and FA3 traits, notably oil content (OC) and percent disease index (PDI), indicating its potential for combining high oil quality with disease resistance. CSFI-17008 showed significant associations with FA2 and FA3, including head diameter (HD), seed yield per plant (SYP), oil yield per plant (OYP), OC and PDI, reflecting a balanced performance across structural, yield and resistance traits (Fig. 4C). Inbreds COSF 6B and CSFI-17017 demonstrated notable associations primarily with FA1 traits such as HD, SYP, PDI, OC and OYP, underscoring their robust performance in key yield and quality attributes.

### Comparative performance of GYT, MGIDI and FAI-BLUP

Partial overlap was observed among the three multi-trait selection indices. COSF 6B was selected by both MGIDI and GYT, whereas CSFI-17024 and CSFI-17008 were identified by both FAI-BLUP and MGIDI (Supplementary Fig. 1). No inbred was common to all three indices, demonstrating the complementary strengths of these approaches in identifying superior genotypes.

### Analysis of SSR markers and molecular clustering patterns

**Fig. 5:**
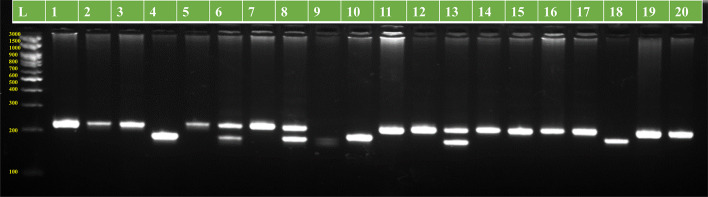
PCR amplification profile of the SSR marker ORS-1245 in 20 sunflower inbred lines. Lane numbers correspond to inbreds as follows: 1 -CSFI 13022, 2 -CSFI 13023, 3-COSF 12B, 4 -CMS 302B, 5 -CMS 335 B, 6-CMS 343 B, 7-PM 81, 8-PS 2023B, 9-CSFI 17001, 10-CSFI 17006, 11-CSFI 17008, 12-CSFI 17017, 13-CSFI 17018, 14-CSFI 17020, 15-CSFI 17024, 16-CSFI 17025, 17COSF 6B, 18-IR 6, 19-COSF 10B, 20 -COSF 12B.

The initial assessment involved screening 141 SSR primers obtained from the literature. Among these, 92 primers exhibited robust amplification, reproducible patterns and multiple polymorphic fragments per assay, whereas the remaining primers were either monomorphic or showed poor amplification. Detailed results, including percentages of polymorphism, Polymorphism Information Content (PIC), Resolving Power (Rp) and Marker Index (MI), are presented in Supplementary Table 4. The screened primers generated well-defined bands, with allele counts ranging from 2 to 6 per locus, resulting in a total of 262 alleles and an average of 2.8 alleles per locus. ORS 898 and ORS 621 had the highest counts of 6 alleles each. All alleles were polymorphic, yielding a 100% polymorphism rate. PCR amplification with SSR marker for 20 sunflower inbreds is shown in Fig. 5. The Polymorphic Information Content (PIC) spanned from 0.170 (ORS 683) to 0.80 (ORS 898), with heterozygosity (H) values of 0.18 and 0.82, respectively. Primer Index (PI) values ranged from 0.19 (ORS 811) to 2.145 (ORS 188) and Resolving Power (Rp) varied from 1.1 (ORS 329) to 7 (ORS 1008). Mean Rp values ranged from 0.37 (ORS 329) to 1.85 (ORS 624). The Multiplex Ratio (MR) was 2.85, while the Marker Index (MI) was 0.95. Cluster analysis using the neighbor-joining method and Jaccard’s pairwise distance matrix identified five distinct clusters among 20 inbred accessions. Clusters I and V each contained five inbreds, clusters II and IV each had three inbreds and cluster III included four inbreds (Fig. 2C). The maximum genetic dissimilarity was 0.69 between IR 6 and COSF15B and the minimum was 0.38 between CSFI17020 and CSFI17024 (Table 3).

**Table 3:** Jaccard Dissimilarity index of 20 inbreds of sunflower.

	1	2	3	4	5	6	7	8	9	10	11	12	13	14	15	16	17	18	19	20
1	1																			
2	0.43	1																		
3	0.56	0.60	1																	
4	0.58	0.59	0.63	1																
5	0.63	0.53	0.62	0.58	1															
6	0.57	0.53	0.62	0.56	0.45	1														
7	0.64	0.61	0.65	0.62	0.58	0.56	1													
8	0.56	0.56	0.62	0.55	0.54	0.41	0.55	1												
9	0.57	0.56	0.58	0.55	0.55	0.45	0.51	0.41	1											
10	0.62	0.58	0.55	0.66	0.57	0.55	0.60	0.51	0.43	1										
11	0.60	0.56	0.63	0.61	0.59	0.55	0.60	0.51	0.52	0.58	1									
12	0.64	0.61	0.66	0.66	0.57	0.65	0.64	0.61	0.58	0.61	0.63	1								
13	0.59	0.59	0.64	0.59	0.58	0.51	0.59	0.53	0.54	0.56	0.58	0.50	1							
14	0.55	0.45	0.63	0.59	0.51	0.49	0.54	0.48	0.46	0.53	0.57	0.57	0.46	1						
15	0.56	0.50	0.60	0.62	0.55	0.53	0.61	0.54	0.47	0.49	0.58	0.61	0.49	**0.38**	1					
16	0.64	0.55	0.57	0.60	0.55	0.53	0.57	0.54	0.48	0.49	0.57	0.60	0.53	0.40	0.43	1				
17	0.60	0.55	0.63	0.64	0.59	0.57	0.61	0.55	0.54	0.58	0.60	0.66	0.50	0.49	0.55	0.52	1			
18	0.67	0.62	**0.69**	0.64	0.64	0.63	0.61	0.65	0.57	0.56	0.67	0.67	0.59	0.57	0.58	0.58	0.56	1		
19	0.57	0.60	0.65	0.66	0.63	0.54	0.66	0.49	0.49	0.55	0.52	0.66	0.59	0.48	0.50	0.49	0.57	0.62	1	
20	0.64	0.59	0.63	0.67	0.60	0.56	0.68	0.62	0.61	0.65	0.56	0.59	0.58	0.61	0.59	0.59	0.62	0.66	0.55	1

The inbreds are follows as: 1. CSFI 13022, 2. CSFI 13023, 3. COSF15B, 4. CMS302B, 5. CMS335B, 6.CMS343 B 7. PM81, 8. PS2023B, 9. CSFI17001, 10. CSFI17006, 11. CSFI17008, 12. CSFI17017, 13. CSFI17018, 14 CSFI17020, 15. CSFI17024, 16. CSFI17025, 17. COSF6B, 18. IR6, 19. COSF10B and 20. COSF12B.

### Principal coordinate analysis (PCoA)

PCoA uses a dissimilarity matrix from allelic data to assess individual and group variances, ascertain outliers and validate genetic relationships. The first three axes (PC1, PC2 and PC3) collectively explain 26.28% of the total variation, contributing 9.66%, 8.62% and 7.99%, respectively (Table 4). All inbreds are positioned noticeably far from the cluster centroid, with PS 2023B, CMS 343B, CSFI 17001, CMS 302B, CSFI 13022, CSFI 17024 and CSFI 17025 being particularly distant (Supplementary Fig. 2).

**Table 4:** Proportion of variation explained by the first three axes of principal coordinate analysis (PCoA) in 20 sunflower inbreds.

S.NO	Axis	1	2	3
1	Percent of variation	9.66	8.62	7.99
2	Cumulative Percent of variation	9.66	18.29	26.28

### Analysis of molecular variance (AMOVA)

The molecular variance within sunflower populations was analyzed using Analysis of Molecular Variance (AMOVA). The results revealed that a substantial of 88% variation was attributed to within-population differences, while only 12% of the variation occurred among the populations (Table 5).

**Table 5:** A summary of molecular variance within and among sunflower populations was evaluated using Analysis of Molecular Variance (AMOVA) in 20 sunflower inbreds.

Source	Df	SS	MS	Est. Var	%
Among Population	4	261.667	65.417	5.966	12
Within Population	15	627.783	41.852	41.852	88
Total	19	889.450		47.818	100

*df* Degree of Freedom, *SS* Sum Square, *MS* Mean sum square, *Est*. *Var* Estimated variance and *%* percentage of variance.

### Model based population structure

**Fig. 6:**
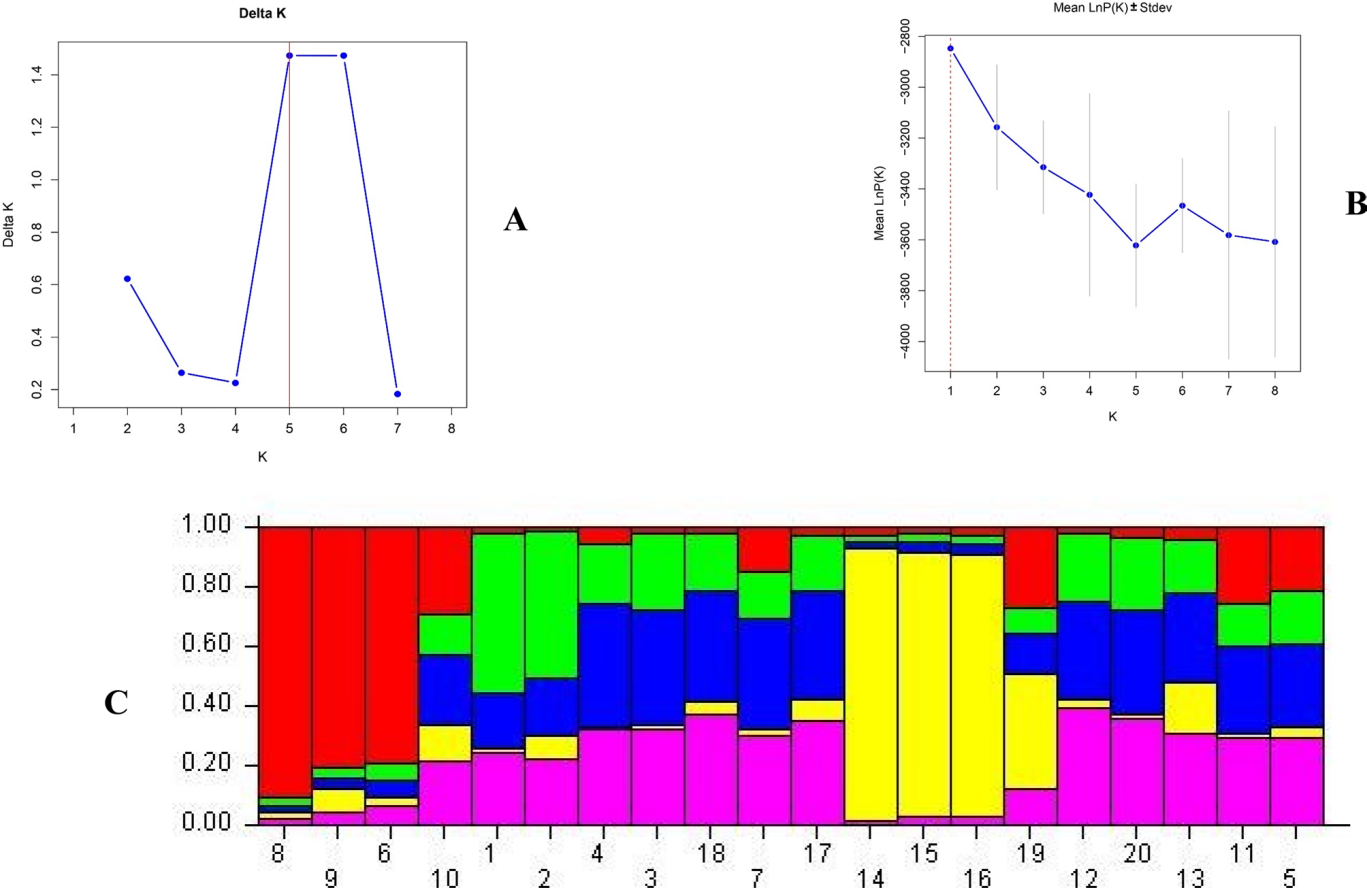
Estimation of hypothetical sub-populations using ΔK-values. (**A**) Delta K, (**B**) Mean LnP(K). (**C**) A model-based population structure plot for 20 inbred lines was generated using STRUCTURE software with K = 5, based on 92 polymorphic SSR markers.

The structure analysis based on SSR marker data grouped the 20 sunflower inbreds into five distinct subpopulations. Bayesian methods and admixture model simulations identified (K = 5) as the optimal number of subpopulations, with the absolute value of the log probability (Ln’'(K)) being 355.64 and the maximum delta K at 1.4736 (Fig. 6A,B). Structure software assessed genetic relatedness, classifying accessions with membership coefficients above 0.80 as genetically pure and those below as intermixed. Among the populations, 30% (six inbreds) were identified as pure, as shown in Fig. 6C. Net nucleotide distances showed the greatest genetic divergence between subpopulations I and IV (0.037) and the lowest between III and V (0.001). Expected heterozygosity was highest in subpopulation III (0.376) and lowest in IV, which also had a low Fst value of 0.3058. The mean Fst value was highest in subpopulation IV (0.2921) and lowest in III (0.0009). Significant divergence among subpopulations is illustrated in Supplementary Fig. 3. The divergence structure is further illustrated through triangular plots and histograms of alpha and Fst (Supplementary Fig. 4). The allelic pattern shows that subpopulation III has a higher number of distributed alleles, while subpopulation II has a lower allelic distribution.

### Molecular characterization of *G. latisporus*

The presence of *G. latisporus* in the sample was confirmed through phase contrast microscopy (Fig. 1A,B). The DNA extracted from the conidia was amplified using the PMITS primer, which amplified at 750 bp product. Amplification of DNA with the G1/G2 primer pair, specific to *G. latisporus*, produced a 418 bp fragment (Fig. 1C).

### Screening for powdery mildew resistance in inbreds

The percent disease index was calculated at 45, 60 and 75 days after sowing (DAS) in which IR6 and PM 81 were used as resistant checks and COSF 15B as susceptible check. In winter 2022, the PDI values ranged from 6.75 (IR6) to 47.74 (COSF 12B), with ten inbreds classified as moderately resistant (PDI 11–25) and five inbreds, including IR6, PM 81, CSFI17008, CSFI17018 and CSFI17020, classified as resistant (PDI 0–10) at 75 DAS. In the rainy season of 2023, PDI values ranged from 3.45 (IR6) to 28.66 (COSF 12B), with 12 inbreds classified as moderately resistant (PDI 11–25) and six inbreds, including IR6, PM 81, CSFI13023, CSFI17008, CSFI17018 and CSFI17020, classified as resistant (PDI 0–10) at 75 DAS, as detailed in Table 6. In *Summer* 2023, the PDI ranged from 5.28 (IR6) to 45.83 (COSF 12B). Among the inbreds, COSF 12B, COSF15B, COSF 10B and PS 2023B were classified as susceptible (S) with PDI values > 25, IR6 had the lowest PDI and was classified as resistant (R) along with PM 81, CSFI17008, CSFI17018 and CSFI17020 were also resistant (R) with PDI values between 0 and 10. Additionally, 11 inbreds were found to be moderately resistant (MR) with PDI values of 10–25.

**Table 6:** Percent disease index of sunflower inbreds across three seasons.

S.No	Inbreds	*Rabi* 2022					*Summer* 2023					*Kharif* 2023				
		PDI45	PDI60	PDI75	Mean	PMR	PDI45	PDI60	PDI75	Mean	PMR	PDI45	PDI60	PDI75	Mean	PMR
1	CSFI 13022	6.28	15.89	20.77	14.31	MR	7.35	18.96	22.84	16.38	MR	3.18	8.23	12.39	7.93	R
2	CSFI 13023	2.92	6.77	19.98	9.89	R	3.69	9.54	21.75	11.66	MR	0.5	4.56	9.63	4.90	R
3	COSF15B	12.08	29.25	42.18	27.84	S	13.72	32.89	44.82	30.48	S	6.25	12.36	25.27	14.63	MR
4	CMS302B	5.68	8.12	22.53	12.11	MR	6.75	8.75	22.16	12.55	MR	2.96	5.25	12.69	6.97	R
5	CMS335B	7.12	14.53	19.41	13.69	MR	8.56	16.97	20.85	15.46	MR	3.56	8.41	13.52	8.50	R
6	CMS343 B	3.58	9.56	16.22	9.79	R	4.75	11.73	17.39	11.29	MR	1.63	6.66	11.36	6.55	R
7	PM81	3.25	5.2	6.75	5.07	R	3.57	5.72	6.27	5.19	R	1.12	3.23	5.56	3.30	R
8	PS2023B	13.25	25.36	33.28	23.96	MR	15.74	31.85	38.77	28.79	S	6.48	15.82	24.63	15.64	MR
9	CSFI17001	10.23	15.41	23.49	16.38	MR	12.57	17.75	24.83	18.38	MR	5.86	12.23	19.63	12.57	MR
10	CSFI17006	5.95	8.06	21.44	11.82	MR	7.78	9.89	22.27	13.31	MR	3.18	6.25	11.72	7.05	R
11	CSFI17008	4.39	6.7	9.28	6.79	R	5.37	7.68	9.58	7.54	R	2.96	5.25	8.63	5.61	R
12	CSFI17017	8.13	12.6	16.03	12.25	MR	8.71	13.18	15.61	12.50	MR	5.28	9.52	12.63	9.14	R
13	CSFI17018	2.36	5.17	9.85	5.79	R	2.15	6.96	9.94	6.35	R	0.18	3.81	5.61	3.20	R
14	CSFI17020	1.95	5.31	9.25	5.50	R	2.17	8.53	9.76	6.82	R	0.4	5.21	8.63	4.75	R
15	CSFI17024	6.45	13.73	16.7	12.29	MR	8.52	15.8	17.77	14.03	MR	3.15	7.13	13.63	7.97	R
16	CSFI17025	9.96	11.76	22.89	14.87	MR	11.92	13.72	23.85	16.50	MR	4.58	9.63	17.63	10.61	MR
17	COSF6B	14.63	19.03	26.47	20.04	MR	13.79	18.19	24.63	18.87	MR	8.26	12.36	19.23	13.28	MR
18	IR6(CHECK)	2.05	5.18	6.95	4.73	R	1.38	4.51	5.28	3.72	R	0.39	2.86	3.45	2.23	R
19	COSF10B	6.96	14.89	38.88	20.24	MR	8.91	16.84	39.83	21.86	MR	4.28	9.53	22.63	12.15	MR
20	COSF12B	15.63	23.82	47.74	29.06	S	17.72	22.91	45.83	28.82	S	8.86	15.53	28.66	17.68	MR

*PDI* Percent disease index, *PMR* Powdery mildew reaction,  *R* Resistant, *MR* Moderate resistant, *S* susceptible.

### Validation of ORS 684 and ORS 1110 SSR markers

Validation of SSR markers viz., ORS 684 and ORS 1110 using Single-marker analysis for powdery mildew disease reaction was performed in 20 inbreds, where ORS 684 and ORS 1110 explained 27.17% and 14.83% of the phenotypic variation explained (PVE%), respectively, with significant adjusted R^2^ values. The marker ORS 684 produced a 175 bp allele for resistant inbreds (IR 6, CSFI 17018, CSFI 17020) and a 165 bp allele for susceptible inbreds (PS 2023B, COSF 10B, COSF 12B, COSF 15B) (Supplementary Fig: 5). The ORS 1110 marker showed a uniform 190 bp allele for all inbreds, failing to differentiate between resistant and susceptible types.

## Discussion

Powdery mildew (*Golovinomyces latisporus*) infection significantly reduces sunflower oil quality and seed yield. While fungicide spraying is common for control, it harms the environment, poses risks to farmers and consumers and raises costs. Breeding programs therefore prioritize identifying and using resistant inbreds to develop high-yielding, resilient cultivars. Evaluating pooled mean performance across seasons helps select superior lines like COSF 6B and COSF 12B, known for their oil content and yield. Transitioning to genetic resistance offers a sustainable, safer and cost-effective alternative to chemical control.

This study comprehensively evaluated sunflower inbreds using three multi-trait selection indices Genotype-by-Yield*Trait (GYT) biplot, Multi-Trait Genotype-Ideotype Distance Index (MGIDI) and Factor Analysis and Ideotype-Based BLUP (FAI-BLUP) to integrate yield, oil quality and disease resistance into the selection process. The GYT biplot standardized phenotypic means by combining oil yield per plant (OYP) with traits where higher values are desirable and dividing by traits where lower values are preferred, effectively resolving scale differences among traits. Singular value decomposition showed that the first two principal components explained 94.08% of the total variation and acute angles between trait vectors indicated strong positive correlations, supporting simultaneous improvement of multiple traits. The "which-won-where" polygon and genotype ranking plots provided intuitive visual insights into inbred performance, removing the need for subjective trait weighting^8,44–47^. COSF 12B and IR6 consistently outperformed other genotypes. COSF 12B exhibited superior yield performance despite being susceptible to powdery mildew, while IR6 demonstrated strong disease resistance coupled with yield. Their proximity to the ideal inbred position emphasizes their potential as elite parental lines for hybrid breeding.

The MGIDI and FAI-BLUP indices further refined inbred selection by integrating factor analysis on BLUP values. MGIDI uniquely allows breeders to specify whether each trait should be increased or decreased, while enabling trait-specific weighting based on economic or breeding priorities^7,48,49^. In both indices, PCA grouped ten key traits into four principal factors (FA1-FA4), capturing trait correlations and reducing dimensionality for better interpretability. Positive SD% were recorded for HD, SYP, OC and OYP, while negative SD% for PDI, DF and DM indicated reduced disease incidence and shorter crop duration. MGIDI provided graphical visualization of inbred strengths and weaknesses, showing how each factor contributes to overall performance. For instance, CSFI-17024 and CSFI-17008 demonstrated strong associations with FA1 and FA3 traits, including OC and PDI, while COSF 6B and CSFI-17017 excelled in FA1 traits, reflecting solid performance in yield and oil-related attributes. At a 20% selection intensity, MGIDI and FAI-BLUP consistently identified COSF 6B, CSFI-17008 and CSFI-17024 as top-performing inbreds, combining high yield, oil quality and disease resistance and showing strong potential as reliable parental lines for sunflower hybrid development. Comparative analysis showed that COSF 6B was selected by both MGIDI and GYT, while CSFI-17024 and CSFI-17008 were identified by both MGIDI and FAI-BLUP, illustrating the complementary strengths of these indices. No inbred was common across all three methods, reinforcing the notion that each index captures distinct yet valuable dimensions of multi-trait performance. Overall, MGIDI stood out as the most robust and informative tool for multi-trait selection due to its customizable trait weighting and clear graphical outputs. The GYT biplot provided intuitive and objective genotype comparisons, while FAI-BLUP offered straightforward rankings. By integrating these complementary approaches, breeders can efficiently identify elite sunflower inbreds with superior yield, oil quality and disease resistance. These methods facilitate strategic hybrid development and marker-assisted backcrossing, streamlining the breeding pipeline toward the development of high-performing, powdery mildew-resistant sunflower cultivars.

Molecular markers, such as microsatellites (SSR), provide a reliable and efficient way to study genetic diversity in plants, surpassing traditional biometrical methods that are influenced by environmental factors. SSRs marker shows stable, consistent insights into parental line dissimilarity, making them a significant improvement over conventional approaches. SSR markers proved effective for parent identification and selection of superior inbreds. They offer more reliable alternative to traditional, environmentally affected grow-out trials for varietal and hybrid identification and inbreds selection, overcoming the challenges of genetic impurity in cross-pollinated crops^2,50,51^. Studying genetic variance is essential for breeders to identify promising inbreds and this investigation employed SSR markers to analyze genetic relationships among sunflower inbreds. Metrics like PI, Rp, EMR and MI evaluate marker efficiency, aiding in the selection of effective markers and primer sets for optimal genetic analysis. Marker utility depends on PIC value, Rp and MI, crucial for genotypic differentiation^52^. In this study, the number of alleles per locus ranged from 2 to 6, with an average of 2.8. Ibrar et al.^19^ reported 1 to 4 alleles per locus with a mean of 2.1, while Antonova et al.^53^ observed an average of 2.2 alleles using 10 SSR markers. Furthermore, Lavudya et al.^2^ identified 267 alleles, with a mean of 2.6 alleles per locus. Primers with the highest PIC values indicated their effectiveness in distinguishing individuals and populations, high PIC values are crucial for selecting informative markers, while markers with low PIC values should be avoided. Based on clustering by using DARWIN v.6.0 software, Clusters I and V had more inbreds, indicating higher genetic diversity, while Clusters II and IV had fewer inbreds, suggesting lower diversity and greater similarity among them. The maximum Jaccard’s coefficient highlights significant genetic dissimilarity, indicating that the inbreds are highly distinct, while a lower coefficient suggests fewer shared alleles and greater genetic divergence. Pandey et al.^54^ and Jannatdoust et al.^55^ also identified three major clusters using hierarchical and neighbor-joining methods, respectively, whereas Lavudya et al.^2^ classified 48 sunflower germplasm accessions into five distinct clusters based on SSR marker data.

The two-dimensional PCoA plot effectively mirrors the groupings, first three axes of the total variation, highlighting their significance for understanding data patterns, though substantial variation. Inbreds distant from the cluster centroid suggest significant genetic differences or outliers, while those near the centroid indicate similarity to the cluster’s average profile. Perveen et al.^56^ reported that the first two principal axes accounted for 18.12% and 12.31% of the variation, respectively, with a cumulative contribution of 30.43%. Hladni et al.^57^ showed that principal coordinates explained 58.4% of the total variability, while Jannatdoust et al.^55^ reported that the first and second components accounted for 7.86% and 6.16% of the variance, respectively. In comparison, Lavudya et al.^2^ reported that the first three principal axes together explained 27.24% of the total variation.

AMOVA analysis of sunflower populations revealed significant variation within populations, primarily attributed to genetic drift and gene flow. The smaller proportion of variation among populations suggests limited genetic differentiation, likely shaped by gene flow, historical migration, or shared ancestry. Gogoi et al.^37^ reported that 87% of the variation occurred within populations and 13% among populations, whereas Jannatdoust et al.^55^ found 70% of the variation within and 30% between populations, highlighting the highly allogamous nature of sunflower. Similarly, Lavudya et al.^2^ observed that most of the variation (89%) was within populations, with only 11% attributed to differences among populations.

The Bayesian method in Structure software effectively analyzed population structure, by allocating inbreds to clusters while maintaining equilibrium within, but not between, clusters. This method helps identify consistent subgroups based on genetic structure, which reflects individual genetics, evolutionary history and gene migration. Molecular diversity is assessed using Darwin and STRUCTURE software, Darwin delve into genetic diversity and relationships through phylogenetic and clustering methods, whereas STRUCTURE focuses on population structure, admixture and individual population assignments. Mandel et al.^58^ reported a maximum of K = 2 clusters in 433 sunflower genotypes, while Ahmed et al.^50^ identified K = 3 subpopulations and Dudhe et al.^59^ detected four populations among 28 sunflower germplasm accessions using 20 SSR markers. Lavudya et al.^2^ grouped 48 sunflower germplasm accessions into five distinct subpopulations (K = 5) based on structure analysis. Six inbreds met the purity threshold, representing 30% of the panel, each subpopulation contained genetically pure germplasms, with net nucleotide distances varying among subpopulations. The fixation index (Fst), ranging from 0 to 1, revealed that subpopulation III exhibited the highest diversity and Fst value, indicating substantial genetic differentiation and limited gene flow. In contrast, subpopulation IV showed the lowest diversity and Fst value, suggesting extensive gene flow and similar genetic characteristics. Mandel et al.^58^ found pairwise Fst values across 12 cultivar classes. Genetic distance between populations plays a crucial role in germplasm conservation, as hybrids from genetically distant parents often exhibit superior traits. Therefore, analyzing population structure is essential for identifying breeding subpopulations^60^. A triangular plot is used to visualize the genetic composition of individuals across subpopulations, with each corner representing a subpopulation and the points indicating the genetic makeup, which highlights admixture and genetic similarity or dissimilarity Lavudya et al.^2^. Histograms of alpha values and Fst values illustrate admixture coefficients and genetic differentiation between subpopulations, respectively. The high allelic distribution in subpopulation III reflects greater genetic diversity, which enhances adaptability, whereas the lower allelic distribution in subpopulation II indicates reduced diversity.

Powdery mildew pathogen (*Golovinomyces latisporus*) is a season-bound, parasitic in nature that can cause unreliable screening results, making molecular markers crucial for marker-assisted breeding^29,31,61^. The PMITS1/PMITS2 primer, which amplifies at 700–800 bp, confirms powdery mildew presence^30,62^. The G1/G2 primer pair, amplifying at 418 bp, identifies *G. cichoracearum*, now known as *Golovinomyces latisporus*^63^. Winter *season* observed a higher PDI, while rainy season had a lower PDI, winter provided more favorable conditions for powdery mildew, resulting in more severe symptoms compared to rainy season. Powdery mildew severity in sunflowers is affected by environmental factors. *Kharif* rainfall can wash away conidia and disrupt mycelium, while a dry spell after rain during the crop’s growth stage can increase disease severity during *rabi* season^64^. In both condition CSFI17008, CSFI17018 and CSFI17020 were classified as resistant (R) with PDI values between 0 and 10, similar to resistant check varieties IR6 and PM 81, these inbreds consistently showed resistance and can be utilized in breeding programs, introgression and marker-assisted breeding. Kulkarni et al.^65^ screened 120 accessions for powdery mildew resistance, finding only 2 restorer lines resistant, 48 accessions medium resistant, 63 susceptible and 7 highly susceptible. Suresha et al.^66^ reported that out of 57 genotypes screened for powdery mildew resistance, 31 were resistant and 25 were susceptible to the disease. Validating linked markers for disease resistance enhances marker-assisted selection^67^. ORS 684 and ORS 1110 were validated through single-marker analysis, demonstrating significant adjusted R^2^ values, with ORS 684 effectively distinguishing resistant from susceptible inbreds and recommended for identifying powdery mildew resistance in breeding programs. Overall morphological data shows that COSF 6B, COSF 10B, COSF 12B and COSF 15B have high seed yield and oil content but are powdery mildew susceptible. In contrast, CSFI 13023, CSFI 17008, CSFI 17018 and CSFI 17020 are resistant but have lower yields. Both resistant and susceptible inbreds can be used in crossing programs and marker-assisted breeding for QTL mapping, fine-mapping to develop powdery mildew resistant inbreds, varieties and hybrids.

## Conclusion

This study identified promising sunflower inbreds for exploitation in breeding programs, combining high yield, oil content and disease resistance. To enhance selection efficiency, three multi-trait selection indices were applied, with the MGIDI index clearly demonstrating its superiority in identifying superior inbred lines. The SSR marker ORS 684 reliably distinguished resistant and susceptible genotypes and explained 27.17% of the phenotypic variation, validating its potential as a foreground marker for marker-assisted breeding. Inbreds such as COSF 6B, COSF 10B, COSF 12B, COSF 15B, CSFI 13023, CSFI 17008, CSFI 17018 and CSFI 17020 emerged as ideal candidates for hybrid development and backcross breeding programs. These findings provide a robust foundation for developing high-yielding, disease-resistant sunflower hybrids, ensuring the effective utilization of genetic resources and accelerating the improvement of elite germplasm.

## Supplementary Information

Below is the link to the electronic supplementary material.

Supplementary Material 1.

## Data Availability

The datasets produced and/or examined during this study can be obtained from the corresponding author upon reasonable request.

## Abbreviations

DF: Days to 50% flowering
DM: Days to Maturity
PH: Plant height
HD: Head diameter
HS: Hundred seed weight
VW: Volume weight per 100 ml
OC: Oil content
SYP: Yield per plant
OYP: Oil yield per plant
PDI: Percent of disease index
