# Supplementary material for: Utilizing multi-trait selection indices for genetic characterization and SSR marker validation linked to powdery mildew resistance in sunflower
Source: Sci Rep. 2026 May 27;16:24256. doi: 10.1038/s41598-026-52132-3 (PMC13444073; doi:10.1038/s41598-026-52132-3)
Supplement: Supplementary file 1 [file 41598_2026_52132_MOESM1_ESM.docx]

**Supplementary table 1: Weather parameter data from the September 2022 to November 2023**

| Year | Month | Max T | Min T | RH M | RH E | RF | EV | SR | SH | WS |
| --- | --- | --- | --- | --- | --- | --- | --- | --- | --- | --- |
| 2022 | September | 31.24 | 22.57 | 76.61 | 54.43 | 1.08 | 5.56 | 329.19 | 6.97 | 6.50 |
| 2022 | October | 30.62 | 22.40 | 84.97 | 57.61 | 3.00 | 4.56 | 263.76 | 5.15 | 3.95 |
| 2022 | November | 29.35 | 21.79 | 85.63 | 58.10 | 4.60 | 4.17 | 278.19 | 4.88 | 3.83 |
| 2022 | December | 28.93 | 20.64 | 85.03 | 53.39 | 3.32 | 4.29 | 292.94 | 5.29 | 4.78 |
| 2023 | January | 29.56 | 19.05 | 84.29 | 39.81 | 0.02 | 4.88 | 365.37 | 7.32 | 5.30 |
| 2023 | February | 32.16 | 18.95 | 79.96 | 27.46 | 0.05 | 8.23 | 387.28 | 8.62 | 5.65 |
| 2023 | March | 33.96 | 22.14 | 80.03 | 33.42 | 0.48 | 7.25 | 375.56 | 8.39 | 6.45 |
| 2023 | April | 35.67 | 23.97 | 80.43 | 37.47 | 1.16 | 6.95 | 358.77 | 8.58 | 5.00 |
| 2023 | May | 34.30 | 24.24 | 87.74 | 56.90 | 5.89 | 5.52 | 333.11 | 7.19 | 4.29 |
| 2023 | June | 33.78 | 24.03 | 83.37 | 50.93 | 0.49 | 6.01 | 343.52 | 6.50 | 5.37 |
| 2023 | July | 31.16 | 23.36 | 83.42 | 57.23 | 1.53 | 5.45 | 300.41 | 4.17 | 11.13 |
| 2023 | August | 33.52 | 23.51 | 83.03 | 48.29 | 0.04 | 6.51 | 364.99 | 7.95 | 7.21 |
| 2023 | September | 32.51 | 23.81 | 83.17 | 54.97 | 0.39 | 5.96 | 314.03 | 4.97 | 8.04 |
| 2023 | October | 32.90 | 23.61 | 84.84 | 46.68 | 34.50 | 5.07 | 322.54 | 6.06 | 4.45 |
| 2023 | November | 29.97 | 22.93 | 91.03 | 61.10 | 391.70 | 3.58 | 312.99 | 5.44 | 4.39 |

Max T: Maximum temperature (°C), Min T: Minimum temperature (°C), RH M: Relative humidity morning (%), RH E: Relative humidity evening (%), RF: Rainfall (mm), EV: Evaporation, SR: Solar radiation, SH: Sunshine hours, WS: Wind speed.

**Supplementary table 2: A list of the twenty sunflower inbreds and their sources used in the genetic diversity study**

| S.no | Inbreds | Source | S.no | Inbreds | Source |
| --- | --- | --- | --- | --- | --- |
| 1 | CSFI 13022 | TNAU, Coimbatore | 11 | CSFI17008 | TNAU, Coimbatore |
| 2 | CSFI 13023 | TNAU, Coimbatore | 12 | CSFI17017 | TNAU, Coimbatore |
| 3 | COSF15B | TNAU, Coimbatore | 13 | CSFI17018 | TNAU, Coimbatore |
| 4 | CMS302B | UAS, Bangalore | 14 | CSFI17020 | TNAU, Coimbatore |
| 5 | CMS335B | UAS, Bangalore | 15 | CSFI17024 | TNAU, Coimbatore |
| 6 | CMS343 B | UAS, Bangalore | 16 | CSFI17025 | TNAU, Coimbatore |
| 7 | PM81 | UAS, Raichur | 17 | COSF6B | TNAU, Coimbatore |
| 8 | PS2023B | UAS, Raichur | 18 | IR6 | IIOR, Hyderabad |
| 9 | CSFI17001 | TNAU, Coimbatore | 19 | COSF10B | TNAU, Coimbatore |
| 10 | CSFI17006 | TNAU, Coimbatore | 20 | COSF12B | TNAU, Coimbatore |

IIOR: Indian institute of oil seed research; TNAU: Tamil Nadu agricultural university and UAS: University of agricultural science.

**Supplementary table 3: Pooled analysis of variance for morphological data across two seasons for all traits in twenty sunflower inbreds**

| Source | **df** | **Mean Sum of Squares** | | | | | | | | |
| --- | --- | --- | --- | --- | --- | --- | --- | --- | --- | --- |
|  |  | **PH** | **DF** | **DM** | **HD** | **SYP** | **VW** | **HSW** | **OC** | **OYP** |
| Replication/ Season | 4 | 10.22 | 12.06 | 22.69 | 0.50 | 2.54 | 3.51 | 0.21 | 0.65 | 0.31 |
| Inbreds | 19 | 1420.93** | 181.56 ** | 131.42 ** | 17.10 * | 82.26 ** | 130.26 ** | 3.36 * | 162.30** | 19.95 ** |
| Season | 1 | 516.92** | 48.13 * | 172.8 ** | 11.36 ** | 349.66 ** | 194.54 ** | 1.81 ** | 15.22 ** | 53.83 ** |
| Inbreds x Season | 19 | 68.80** | 42.38 ** | 40.97 ** | 6.95 ** | 19.20 ** | 11.61 * | 1.49 ** | 12.83 ** | 3.005 ** |
| Error | 76 | 28.31 | 8.98 | 12.83 | 0.81 | 6.66 | 6.73 | 0.22 | 1.46 | 0.98 |

DF:Days to 50% flowering, DM:Days to Maturity, PH:Plant height, HD:Head diameter, HS:Hundred Seed weight VW: Volume weight per 100ml, OC: Oil content, SYP: Yield per plant and OYP: Oil yield per plant.

**Supplementary table 4: Genotyping data using SSR markers across the studied sunflower inbreds.**

| S.no | Marker | A | H | PIC | PI | RP | Mean RP |
| --- | --- | --- | --- | --- | --- | --- | --- |
| 1 | ORS 1265 | 4 | 0.51 | 0.47 | 0.495 | 2.3 | 0.58 |
| 2 | ORS 317 | 2 | 0.36 | 0.29 | 0.775 | 1.7 | 0.85 |
| 3 | ORS 1159 | 4 | 0.74 | 0.69 | 1.71 | 3.6 | 0.90 |
| 4 | ORS 878 | 3 | 0.64 | 0.56 | 1.22 | 1.8 | 0.60 |
| 5 | ORS 7 | 3 | 0.57 | 0.49 | 1.05 | 1.6 | 0.53 |
| 6 | ORS 605 | 4 | 0.45 | 0.35 | 0.915 | 1.7 | 0.85 |
| 7 | ORS 1045 | 4 | 0.47 | 0.44 | 0.57 | 2 | 0.50 |
| 8 | ORS 658 | 3 | 0.44 | 0.38 | 0.895 | 1.7 | 0.57 |
| 9 | ORS 124 | 2 | 0.47 | 0.363 | 0.915 | 2.3 | 1.15 |
| 10 | ORS 924 | 3 | 0.53 | 0.44 | 0.77 | 3 | 1.00 |
| 11 | ORS 488 | 2 | 0.23 | 0.2 | 0.635 | 1.5 | 0.75 |
| 12 | ORS 898 | 6 | 0.82 | 0.8 | 0.86 | 2.4 | 0.40 |
| 13 | ORS 202 | 3 | 0.54 | 0.44 | 0.37 | 4 | 1.33 |
| 14 | ORS 683 | 2 | 0.18 | 0.17 | 0.435 | 1.9 | 0.95 |
| 15 | ORS 935 | 2 | 0.37 | 0.3 | 0.75 | 2 | 1.00 |
| 16 | ORS 1288 | 3 | 0.57 | 0.51 | 1.16 | 2.4 | 0.80 |
| 17 | ORS 785 | 2 | 0.62 | 0.54 | 0.51 | 3.4 | 1.70 |
| 18 | ORS 644 | 2 | 0.3 | 0.26 | 0.875 | 2.1 | 1.05 |
| 19 | ORS 337 | 2 | 0.5 | 0.3 | 0.875 | 2.1 | 1.05 |
| 20 | ORS 200 | 3 | 0.49 | 0.43 | 0.695 | 2.1 | 0.70 |
| 21 | ORS 341 | 3 | 0.57 | 0.52 | 1.155 | 2.1 | 0.70 |
| 22 | ORS 620 | 3 | 0.33 | 0.31 | 0.19 | 2.2 | 0.73 |
| 23 | ORS111 | 2 | 0.25 | 0.22 | 0.51 | 2 | 1.00 |
| 24 | ORS1120 | 2 | 0.4 | 0.32 | 0.83 | 1.8 | 0.90 |
| 25 | ORS 1024 | 3 | 0.62 | 0.55 | 1.33 | 2.6 | 0.87 |
| 26 | ORS 530 | 3 | 0.48 | 0.36 | 1.215 | 2.3 | 0.77 |
| 27 | ORS237 | 2 | 0.49 | 0.37 | 0.98 | 1.8 | 0.90 |
| 28 | ORS 484 | 4 | 0.7 | 0.64 | 1.17 | 5 | 1.25 |
| 29 | ORS 725 | 5 | 0.76 | 0.72 | 1.585 | 6.3 | 1.26 |
| 30 | ORS 1193 | 4 | 0.71 | 0.66 | 1.44 | 3.8 | 0.95 |
| 31 | ORS 1259 | 2 | 0.38 | 0.31 | 0.795 | 1.9 | 0.95 |
| 32 | ORS 323 | 4 | 0.55 | 0.5 | 0.475 | 3.3 | 0.83 |
| 33 | ORS 700 | 2 | 0.34 | 0.28 | 0.74 | 1.8 | 0.90 |
| 34 | ORS 966 | 2 | 0.53 | 0.44 | 0.935 | 2.9 | 0.97 |
| 35 | ORS 604 | 4 | 0.72 | 0.67 | 1.625 | 3.9 | 0.98 |
| 36 | ORS 1013 | 2 | 0.19 | 0.17 | 0.5 | 1.8 | 0.90 |
| 37 | ORS 1043 | 3 | 0.4 | 0.32 | 0.82 | 1.4 | 0.70 |
| 38 | ORS 329 | 2 | 0.56 | 0.47 | 0.835 | 1.1 | 0.37 |
| 39 | ORS 510 | 2 | 0.38 | 0.31 | 0.795 | 1.9 | 0.95 |
| 40 | ORS 887 | 2 | 0.5 | 0.37 | 1 | 2 | 1.00 |
| 41 | ORS 882 | 2 | 0.46 | 0.35 | 0.87 | 2.4 | 1.20 |
| 42 | ORS 795 | 3 | 0.46 | 0.35 | 0.935 | 1.9 | 0.95 |
| 43 | ORS 506 | 3 | 0.58 | 0.5 | 0.875 | 3.7 | 1.23 |
| 44 | ORS 938 | 5 | 0.57 | 0.51 | 0.98 | 2.2 | 0.44 |
| 45 | ORS 188 | 5 | 0.79 | 0.76 | 2.145 | 5.3 | 1.06 |
| 46 | ORS 684 | 3 | 0.51 | 0.45 | 0.815 | 2.7 | 0.90 |
| 47 | ORS 1110 | 2 | 0.43 | 0.33 | 0.875 | 1.9 | 0.95 |
| 48 | ORS 591 | 4 | 0.42 | 0.39 | 0.685 | 2.3 | 0.58 |
| 49 | ORS1008 | 5 | 0.77 | 0.73 | 1.23 | 7 | 1.40 |
| 50 | ORS 1129 | 2 | 0.29 | 0.24 | 0.675 | 1.7 | 0.85 |
| 51 | ORS 595 | 3 | 0.6 | 0.52 | 1.055 | 1.5 | 0.50 |
| 52 | ORS 818 | 3 | 0.56 | 0.48 | 1.1 | 1.8 | 0.60 |
| 53 | ORS 613 | 4 | 0.38 | 0.35 | 0.7 | 2.2 | 0.55 |
| 54 | ORS 682 | 3 | 0.64 | 0.57 | 1.325 | 2.1 | 0.70 |
| 55 | ORS 990 | 2 | 0.45 | 0.35 | 0.855 | 2.3 | 1.15 |
| 56 | ORS 621 | 6 | 0.78 | 0.75 | 1.69 | 5.9 | 0.98 |
| 57 | ORS228 | 4 | 0.68 | 0.62 | 1.05 | 2 | 0.50 |
| 58 | ORS 686 | 3 | 0.66 | 0.59 | 1.475 | 3.3 | 1.10 |
| 59 | ORS817 | 2 | 0.42 | 0.33 | 0.87 | 1.6 | 0.80 |
| 60 | ORS502 | 3 | 0.51 | 0.44 | 1.055 | 2.1 | 0.70 |
| 61 | ORS 946 | 2 | 0.41 | 0.32 | 0.71 | 2.4 | 1.20 |
| 62 | ORS 358 | 2 | 0.47 | 0.36 | 0.675 | 2.9 | 1.45 |
| 63 | ORS 1009 | 4 | 0.48 | 0.45 | 0.45 | 2.6 | 0.52 |
| 64 | ORS 191 | 3 | 0.45 | 0.41 | 0.725 | 2.7 | 0.90 |
| 65 | ORS 200 | 2 | 0.19 | 0.17 | 0.5 | 1.8 | 0.90 |
| 66 | ORS 418 | 2 | 0.42 | 0.33 | 0.495 | 2.9 | 1.45 |
| 67 | ORS 70 | 2 | 0.42 | 0.46 | 1.38 | 2.6 | 0.65 |
| 68 | ORS215 | 2 | 0.51 | 0.44 | 0.935 | 2.1 | 0.70 |
| 69 | ORS 1079 | 2 | 0.42 | 0.37 | 0.95 | 2.4 | 1.20 |
| 70 | ORS1086 | 3 | 0.42 | 0.33 | 0.775 | 2.3 | 1.15 |
| 71 | ORS706 | 2 | 0.56 | 0.47 | 1.155 | 2.3 | 0.77 |
| 72 | ORS782 | 2 | 0.44 | 0.34 | 0.9 | 1.8 | 0.90 |
| 73 | ORS832 | 3 | 0.46 | 0.35 | 0.92 | 1.6 | 0.80 |
| 74 | ORS624 | 2 | 0.49 | 0.37 | 0.255 | 3.7 | 1.85 |
| 75 | ORS1028 | 2 | 0.6 | 0.53 | 1.25 | 2.6 | 0.87 |
| 76 | ORS1037 | 2 | 0.44 | 0.34 | 0.9 | 1.8 | 0.90 |
| 77 | ORS1242 | 3 | 0.47 | 0.36 | 0.935 | 2.1 | 1.05 |
| 78 | ORS585 | 3 | 0.48 | 0.36 | 0.96 | 2 | 1.00 |
| 79 | ORS812 | 2 | 0.43 | 0.36 | 0.77 | 2.4 | 0.80 |
| 80 | ORS216 | 3 | 0.49 | 0.37 | 0.415 | 3.5 | 1.75 |
| 81 | ORS1271 | 3 | 0.65 | 0.58 | 1.45 | 3.2 | 1.07 |
| 82 | ORS703 | 4 | 0.47 | 0.4 | 0.95 | 1.8 | 0.60 |
| 83 | ORS1212 | 2 | 0.74 | 0.69 | 1.65 | 4.8 | 1.20 |
| 84 | ORS656 | 2 | 0.34 | 0.28 | 0.555 | 2.3 | 1.15 |
| 85 | ORS803 | 2 | 0.49 | 0.37 | 0.83 | 2.8 | 1.40 |
| 86 | ORS996 | 2 | 0.37 | 0.3 | 0.75 | 2 | 1.00 |
| 87 | ORS801 | 3 | 0.34 | 0.32 | 0.75 | 2 | 1.00 |
| 88 | ORS1245 | 2 | 0.53 | 0.42 | 1.045 | 2.5 | 0.83 |
| 89 | ORS811 | 2 | 0.09 | 0.09 | 0.19 | 2 | 1.00 |
| 90 | ORS628 | 4 | 0.48 | 0.36 | 0.955 | 1.7 | 0.85 |
| 91 | ORS461 | 3 | 0.7 | 0.65 | 1.05 | 5.4 | 1.35 |
| 92 | ORS415 | 2 | 0.44 | 0.38 | 0.89 | 2 | 0.67 |
|  | Minimum | 2 | 0.09 | 0.09 | 0.19 | 1.1 | 0.37 |
|  | Maximum | 6 | 0.82 | 0.8 | 2.145 | 7 | 1.85 |
|  | Average | 2.84 | 0.49 | 0.42 | 0.91 | 2.55 | 0.93 |

A: Number of allele, H: heterozygosity, PIC: Polymorphic Information Content, PI: Primer Index, (Rp):Resolving Power.

**Supplementary fig 1:** **Venn Diagram Showing Overlap of Selected Hybrids by GYT, MGIDI, and FAI-BLUP Methods.**

Principal Coordinates (PCoA)

| Coord. 1 | Coord. 2 |
| --- | --- |
| 0.40112 | -0.763642 |
| 0.595059 | 1.03594 |
| -0.341713 | 0.570663 |
| -0.679068 | 0.787155 |
| -0.574839 | 0.992855 |
| -1.43542 | 0.168101 |
| -1.44313 | 0.613156 |
| -1.66285 | 0.299737 |
| 1.64264 | 0.783538 |
| 0.824716 | 1.02119 |
| 1.1311 | 0.940689 |
| 1.82328 | -0.579459 |
| 0.582037 | -0.616963 |
| -1.05869 | -0.367914 |
| 0.661081 | 0.792666 |
| 0.619013 | -0.398272 |
| 0.27325 | -1.80971 |
| 0.0462193 | -2.09229 |
| -0.532448 | -1.29524 |
| -0.871359 | -0.082191 |

**Supplementary fig 2: Performing principal coordinate analysis on sunflower genotypes using SSR markers data.**

**Supplementary fig 3: Significant divergence (Fst) is evident among sub-populations. A) Fst1 vs Iterations, B) Fst2 vs Iterations, C) Fst3 vs Iterations, D) Fst4 vs Iterations and E) Fst5 vs Iterations**

**Supplementary fig 4: Plotting the structure of significant divergence among sub-population. (A) Triangular plot, (B) Histogram distribution of Alpha.**

| **L** | **1** | **2** | **3** | **4** | **5** | **6** | **7** | **8** | **9** | **10** | **11** | **12** | **13** | **14** | **15** | **16** | **17** | **18** | **19** | **20** |
| --- | --- | --- | --- | --- | --- | --- | --- | --- | --- | --- | --- | --- | --- | --- | --- | --- | --- | --- | --- | --- |

**Supplementary fig 5: Molecular profiling of the reported markers for ORS 684 (LG 10) for twenty inbreds (**The inbreds are follows as: 1 – CSFI 13022, 2 – CSFI 13023, 3 – COSF 12B, 4 – CMS 302B, 5 – CMS 335 B, 6 – CMS 343 B, 7 – PM 81, 8 – PS 2023B, 9 – CSFI 17001, 10 – CSFI 17006, 11 – CSFI 17008, 12 – CSFI 17017, 13 – CSFI 17024, 14 – CSFI 17018, 15 –CSFI 17020, 16 – CSFI 17025, 17 – COSF 6B, 18 – IR 6, 19 – COSF 10B, 20 – COSF 15B)

**Original gel pictures**

Figure 1: Confirmation of powdery mildew pathpgen in sunflower.
A) Chains of conidia observed on leaves using phase contrast microscopy;
B) Conidia visualized in sucrose suspension with phase contrast microscopy;
C) PCR analysis confirming powdery mildew with specific primers-PMITS1/PMITS2 producing a 750 bp fungal band and G1/G2 yielding a 418 bp band specific for *Golovinomyces latisporus.*

Figure 5. PCR amplification profile of the SSR marker ORS-1245 in 20 sunflower inbred lines. Lane numbers correspond to inbreds as follows: 1 -CSFI 13022, 2 -CSFI 13023, 3 -COSF 12B, 4 -CMS 302B, 5 -CMS 335 B, 6 - CMS 343 B, 7 - PM 81, 8 - PS 2023B, 9 - CSFI 17001, 10 - CSFI 17006, 11 - CSFI 17008, 12 - CSFI 17017, 13 - CSFI 17018, 14 - CSFI 17020, 15 - CSFI 17024, 16 - CSFI 17025, 17 - COSF 6B, 18 - IR 6, 19 - COSF 10B, 20 -COSF 12B.

Supplementary fig 5: Molecular profiling of the reported markers for ORS 684 (LG 10) for twenty inbreds (The inbreds are follows as: 1 – CSFI 13022, 2 – CSFI 13023, 3 – COSF 12B, 4 – CMS 302B, 5 – CMS 335 B, 6 – CMS 343 B, 7 – PM 81, 8 – PS 2023B, 9 – CSFI 17001, 10 – CSFI 17006, 11 – CSFI 17008, 12 – CSFI 17017, 13 – CSFI 17024, 14 – CSFI 17018, 15 –CSFI 17020, 16 – CSFI 17025, 17 – COSF 6B, 18 – IR 6, 19 – COSF 10B, 20 – COSF 15B)
